# Severity of coronary atherosclerosis and stroke incidence in 7-year follow-up

**DOI:** 10.1007/s00415-013-6892-4

**Published:** 2013-03-20

**Authors:** Wojciech Sobiczewski, Marcin Wirtwein, Ewelina Trybala, Marcin Gruchala

**Affiliations:** 1 I Department of Cardiology, Medical University of Gdansk, Debinki, 780-211 Gdansk, Poland; 2 Department of Pharmacology, Medical University of Gdansk, Debowa, 2380-211 Gdansk, Poland; 3 Department of Neurology, Specialized Hospital of Chojnice, Lesna 10, 89-600 Chojnice, Poland

**Keywords:** Stroke, Coronary artery disease, Atherosclerosis, Agiography

## Abstract

The purpose of this prospective study was to investigate the association between the severity of coronary atherosclerosis in angiography and the risk of stroke in symptomatic coronary artery disease (CAD) patients without atrial fibrillation or atrial flutter. Associations between stroke and coronary artery disease were examined in 1,183 subjects without a history of stroke and who were referred for diagnostic coronary angiography. Association between stoke and coronary artery disease was determined using the COX proportional hazard regression model. During the follow-up period (mean 6.7 years), 50 strokes occurred. In the group with strokes there was a higher prevalence of multi-vessel coronary artery disease (62 vs. 46 %, *p* < 0.01). In the COX proportional hazard regression model, multi-vessel CAD was significantly associated with the stroke hazard ratio (HR) of 1.8 (CI 1.03–3.43), determined from a 7-year period of observation. Symptomatic patients with multi-vessel CAD are thus at a high risk of stroke development.

## Introduction

Recently, an association between strokes and risk of coronary artery disease (CAD) has been reported [1–3]. However, these studies were performed among patients with symptoms of acute ischemic stroke or transient ischemic attack, and CAD was confirmed on the basis of computed tomography (CT). The gold standard for assessment of coronary artery stenosis is still conventional coronary angiography, which has a higher sensitivity and specificity for detecting coronary lesions [4]. Apart from atherosclerosis, the major causes of ischemic stroke are supraventricular arrhythmias such as atrial fibrillation or atrial flutter. The risk of ischemic stroke in patients with arrhythmias has been well established. But so far, the association between the severity of CAD assessed by angiography and the prevalence of non-arrhythmic-dependent stroke in symptomatic CAD patients has not been investigated. Thus, the present prospective study evaluated the relationship between the severity of coronary atherosclerosis in angiography and the risk of stroke in symptomatic CAD patients without atrial fibrillation or atrial flutter.

## Methods

The study recruited 1,183 consecutive individuals (between August 2003 and August 2006) based on clinical data or the findings of an abnormal stress test, who were referred for diagnostic coronary angiography. The exclusion criteria were previous stroke or transient ischemic attack, atrial fibrillation or atrial flutter [patients with paroxysmal atrial fibrillation were excluded on the basis of the history of this arrhythmia or any evidence in ECG (ECG at rest or Holter ECG), history of any supraventricular and ventricular arrhythmia lasted ≥ 48 h] and other chronic diseases leading to limited life expectancy. The study protocol was approved by the Ethics Committee of the Medical University of Gdansk.

On admission day, before coronary angiography, fasting blood samples were collected in order to measure total cholesterol, HDL cholesterol, LDL cholesterol, and triglycerides levels.

According to the findings of the coronary angiography, significant CAD was considered present when there was ≥70 % coronary artery stenosis (CAS) of at least one coronary artery. The subjects were divided into subgroups: those without CAS, those with single-vessel CAD (one CAS ≥ 70 %) and those with multi-vessel CAD (at least two CAS ≥ 70 %).

Office blood pressure measurement was performed (OMRON 705 IT) and 24-h ambulatory BP monitoring was obtained (SpaceLabs 90210) with BP readings set at 20-min intervals (06:00 AM–06:00 PM) and at 30-min intervals (06:00 PM–06:00 AM).

The subjects were followed from the dates of coronary angiography until December 31, 2011. The follow-up was performed during clinic visits, except for patients who were not able to visit the clinic; in this case the follow-up was performed by phone . Data about stroke diagnosis was verified by review of discharge cards, and if the hospital discharge cards were not available it was verified by information from the National Polish Health Service. Stroke diagnosis was performed by neurologists according to European Stroke Organization guidelines [5]. All subjects were hospitalized in neurologic units, in which stroke was confirmed via computed tomography, but only 21 patients (42 %) underwent magnetic resonance imaging (MRI). The ultrasonography of extracranial arteries was additionally performed in 32 subjects (64 %).

The primary outcomes were all causes of death and were analyzed as “survival free of stroke”. Stroke-free survival was estimated using the Kaplan–Meier curve at 7 years. The Kaplan–Meier analysis was performed after excluding patients who died as a result of the initial stroke. Death related to initial stroke was defined as death within 1 month of stroke onset. Data from the groups of subjects were compared using the Student *t* test for unpaired observations and the χ^2^ test of proportions. Average differences in the groups of CAD were compared by analysis of variance (ANOVA) with post hoc Tukey HSD test. The relative hazard (HR) and 95 % CI were estimated for multi-vessel CAD using COX proportional hazard regression, adjusted for sex, age, HDL cholesterol, use of medications (acetylsalicylic acid, lipid-lowering drugs [statins and/or fibrates], angiotensin converting enzyme inhibitors and others (angiotensin receptor blockers, β-blockers, diuretics and calcium channel blockers) and 24-h systolic BP. Throughout the text, the symbol ± refers to the SD of the mean and *p* < 0.05 was taken as the level of statistical significance.

## Results

During the follow-up period (mean 6.7 years), 50 stroke events occurred in the studied group (7 hemorrhagic and 43 ischemic). According to TOAST classification (Trial of Org 10172 in Acute Stroke Treatment), 18 (42 %) patients had ischemic stroke due to small-vessel occlusion, 10 (23 %) patients had ischemic stroke due to large-vessel atherosclerosis and 15 (35 %) patients had ischemic stroke due to undetermined etiology. None of the patients had ischemic stroke due to cardioembolism or due to nonatherosclerotic vasculopathies or other determined etiology. Among 32 subjects who underwent ultrasonography of extracranial arteries, only 10 (20 %) had ≥60 % stenosis of extracranial arteries. The mean period from inclusion up to stroke was 3.7 years. Within five years of observation, 32 (64 %) strokes occurred. In the 7-year follow-up period, 228 (19 %) patients had coronary angioplasty but none of them were associated with stroke complications (for all patients the period between coronary interventions and stroke was over 30 days).

Table 1 shows baseline characteristics of the cohort group. In patients with developed stroke, CAD with significant CAS was revealed more often with a higher prevalence of multi-vessel CAD in comparison to patients without stroke in the follow-up period.

**Table 1:** Baseline characteristics of the total and study groups

	Total group (*n* = 1,183)	No stroke (*n* = 1,133)	Stroke (*n* = 50)	*p*
Male	720 (60 %)	683 (60 %)	37 (73 %)	NS
Age (years)	63.1 ± 9.2	63.0 ± 9.2	65.9 ± 8.6	0.02
Hypertension	913 (77 %)	870 (77 %)	43 (86 %)	NS
Diabetes	248 (21 %)	237 (21 %)	11 (22 %)	NS
Smokers	182 (15 %)	168 (15 %)	14 (28 %)	<0.01
Total cholesterol (mg/dL)	205.7 ± 53.2	206.0 ± 53.6	197.0 ± 44.5	NS
LDL cholesterol (mg/dL)	121.0 ± 43.1	121.3 ± 43.2	115.2 ± 41.3	NS
HDL cholesterol (mg/dL)	55.1 ± 13.3	55.2 ± 13.4	52.3 ± 12.6	NS
Triglycerides (mg/dL)	147.8 ± 98.2	147.7 ± 98.5	150.3 ± 93.0	NS
Medications				
ASA	925 (78 %)	881 (78 %)	44 (88 %)	0.05
Lipid-lowering drugs	1,080 (91 %)	1,035 (91 %)	45 (90 %)	NS
β-blockers	927 (78 %)	882 (78 %)	45 (90 %)	0.02
Calcium channel blockers	290 (24 %)	278 (24 %)	12 (24 %)	NS
ACE-inhibitors	868 (73 %)	828 (73 %)	40 (80 %)	NS
ARB	50 (4 %)	48 (4 %)	2 (4 %)	NS
Diuretics	222 (19 %)	210 (19 %)	12 (24 %)	NS
Office SBP (mmHg)	137.2 ± 19.9	137.1 ± 20.0	139.8 ± 17.7	NS
Office DBP (mmHg)	78.1 ± 11.2	78.1 ± 11.2	78.3 ± 11.0	NS
Office PP (mmHg)	59.15 ± 15.4	59.1 ± 15.6	61.2 ± 14.7	NS
Office HR (bpm)	70.4 ± 12.1	70.4 ± 12.1	71.4 ± 12.1	NS
24-h systolic BP (mmHg)	123.8 ± 13.6	123.7 ± 13.6	127.4 ± 14.1	NS
24-h diastolic BP (mmHg)	71.3 ± 8.2	71.2 ± 8.2	72.8 ± 8.9	NS
24-h PP (mmHg)	52.5 ± 11.2	52.4 ± 11.2	54.9 ± 11.0	NS
24-h HR (bpm)	66.8 ± 9.7	66.7 ± 9.5	68.1 ± 12.1	NS
CAD	772 (60 %)	806 (71 %)	42 (84 %)	<0.05
Single vessel CAD	271(21 %)	282 (25 %)	11 (22 %)	NS
Multi-vessel CAD	501 (39 %)	524 (46 %)	31 (62 %)	<0.01
CV events (except stroke)	142 (12 %)	113 (10 %)	29 (58 %)	<0.01
Stroke deaths	10 (1 %)	–	10 (20 %)	–
All cause deaths	144 (11 %)	123 (11 %)	20 (40 %)	<0.01

*ASA* acetylsalicylic acid, lipid lowering drugs include statins and fibrates, *ACE* angiotensin converting enzyme, *ARB* angiotensin receptor blocker, *BP* blood pressure, *CAD* coronary artery disease (at least one ≥70 % coronary artery stenosis), *CV* cardiovascular

Table 2 presents the prevalence of stroke and risk factors of atherosclerosis in the subgroups with single-vessel and multi-vessel CAD in comparison to the control subgroup without CAS. In the latter subgroup, the prevalence of stroke was three times lower than in the multi-vessel CAD subgroup.

**Table 2:** Clinical characteristics and prevalence of CV events in the patients without CAS, with single-vessel and multi-vessel CAD

	No CAS (*n* = 335)	Single-vessel CAD (*n* = 293)	Multi-vessel CAD (*n* = 555)
Male	136 (41 %)	192 (65 %)*	392 (71 %)*
Age (years)	61.9 ± 8.6	62.9 ± 9.6	64.0 ± 9.2
Hypertension	258 (77 %)	222 (76 %)	433 (78 %)
Diabetes	52 (15 %)	63 (21 %)	133 (24 %)
Smokers	36 (11 %)	58 (20 %)	88 (16 %)
Total cholesterol (mg/dL)	203.8 ± 48.2	205.7 ± 51.3	206.8 ± 57.1
LDL cholesterol (mg/dL)	118.9 ± 40.6	121.1 ± 42.0	122.3 ± 45.0
HDL cholesterol (mg/dL)	58.7 ± 14.2	54.9 ± 14.1*	53.1 ± 11.9*
Triglycerides (mg/dL)	137.5 ± 93.3	143.8 ± 86.6	156.0 ± 106.1
Medications			
ASA	159 (47 %)	269 (92 %)*	497 (89 %)*
Lipid-lowering drugs	295 (88 %)	266 (91 %)	519 (93 %)
β-blockers	236 (70 %)	232 (79 %)*	459 (83 %)*
Calcium channel blockers	110 (33 %)	63 (21 %)	117 (21 %)
ACE-inhibitors	215 (64 %)	218 (74 %)*	435 (78 %)*
ARB	23 (7 %)	15 (5 %)	12 (2 %)
Diuretics	58 (17 %)	51 (17 %)	113 (20 %)
Office SBP (mmHg)	134.3 ± 18.4	139.5 ± 20.5	137.8 ± 20.3
Office DBP (mmHg)	78.7 ± 10.4	78.6 ± 10.8	77.5 ± 11.8
Office PP (mmHg)	55.5 ± 14.7	60.9 ± 16.1*	60.3 ± 15.5*
Office HR (bpm)	71.5 ± 12.1	69.9 ± 12.2	70.1 ± 11.9
24-h systolic BP (mmHg)	122.0 ± 13.1	124.3 ± 13.2	124.7 ± 14.1^†^
24-h diastolic BP (mmHg)	71.1 ± 8.0	71.3 ± 7.6	71.4 ± 8.7
24-h PP (mmHg)	50.9 ± 10.7	52.9 ± 11.3	53.3 ± 11.4*
24-h HR (bpm)	68.1 ± 9.7	65.8 ± 9.6*	66.5 ± 9.6^†^
Stroke	8 (2 %)	11 (4 %)	31 (6 %)^†^
CV events (except stroke)	27 (8 %)	38 (13 %)^†^	77 (14 %)^†^
Stroke deaths	1 (0.3 %)	2 (0.7 %)	7 (1.3 %)
All cause deaths	25 (7 %)	31 (11 %)	87 (16 %)*

*ASA* acetylsalicylic acid, lipid lowering drugs include statins and fibrates, *ACE* angiotensin converting enzyme, *ARB* angiotensin receptor blocker, *BP* blood pressure, *CAS* coronary artery stenosis, *CAD* coronary artery disease (at least one ≥70 % coronary artery stenosis), *CV* cardiovascular

* p < 0.01 vs. No CAS

^†^ *p* < 0.02 vs. No CAS

In the COX proportional hazard regression model, multi-vessel CAD was significantly associated with stroke HR 1.8 (CI 1.03–3.43).

## Discussion

Many clinical studies have revealed significant association between stroke and CAD in patients with ischemic stroke. All studies have used CT for coronary artery stenosis evaluation, and stenosis of 50 % was considered as significant. The disadvantage of these studies was a low sensitivity and specificity for CT to detect coronary lesions ≤50 %. None of the studies used coronary angiography (the gold standard for coronary artery lesions assessment) for coronary artery stenosis evaluation. Moreover, there were no prospective trials with stroke risk assessment in the follow-up period for patients with angiographically established CAD. In some studies patients with supraventricular arrhythmias (also not associated with CAD) were included.

In the present prospective study, in symptomatic patients with CAD assessed in coronary angiography, significant coronary atherosclerosis (and especially multi-vessel CAD) was significantly associated with the development of stroke in the 7-year follow-up period. In spite of comprehensive treatment of risk factors for atherosclerosis, the risk of stroke in multi-vessel CAD was higher than in patients with no CAS. Patients with multi-vessel CAD were treated more extensively with acetylsalicylic acid, beta blockers and ACE inhibitors than those without coronary atherosclerosis.

The limitation of this study is that the grade of carotid atheroma was not considered in all patients. Because the purpose of this study was not assessment of the relationship between CAD and carotid artery stenosis, only patients with symptoms of carotid stenosis had Doppler ultrasonography performed. Another limitation is that stroke was established mainly on the basis of computed tomography. During the follow-up period, only some of the neurological centers had MR devices.

In conclusion, significant CAD is associated with stroke based on over 7 years of observation. Symptomatic patients with multi-vessel CAD are considered to be at high risk of stroke development.
